# Lymphatic Clearance of Immune Cells in Cardiovascular Disease

**DOI:** 10.3390/cells10102594

**Published:** 2021-09-29

**Authors:** Christophe Ravaud, Nikita Ved, David G. Jackson, Joaquim Miguel Vieira, Paul R. Riley

**Affiliations:** 1 Burdon-Sanderson Cardiac Science Centre, Department of Physiology, Anatomy and Genetics, University of Oxford, Oxford OX1 3PT, UK; christophe.ravaud@dpag.ox.ac.uk (C.R.); nikita.ved@dpag.ox.ac.uk (N.V.); joaquim.vieira@dpag.ox.ac.uk (J.M.V.); 2 MRC Human Immunology Unit, MRC Weatherall Institute of Molecular Medicine, John Radcliffe Hospital, University of Oxford, Oxford OX3 9DS, UK; david.jackson@imm.ox.ac.uk

**Keywords:** lymphangiogenesis, myocardial infarction, immune cells, lymphatic, cell clearance, VEGF-C, LYVE1

## Abstract

Recent advances in our understanding of the lymphatic system, its function, development, and role in pathophysiology have changed our views on its importance. Historically thought to be solely involved in the transport of tissue fluid, lipids, and immune cells, the lymphatic system displays great heterogeneity and plasticity and is actively involved in immune cell regulation. Interference in any of these processes can be deleterious, both at the developmental and adult level. Preclinical studies into the cardiac lymphatic system have shown that invoking lymphangiogenesis and enhancing immune cell trafficking in ischaemic hearts can reduce myocardial oedema, reduce inflammation, and improve cardiac outcome. Understanding how immune cells and the lymphatic endothelium interact is also vital to understanding how the lymphatic vascular network can be manipulated to improve immune cell clearance. In this Review, we examine the different types of immune cells involved in fibrotic repair following myocardial infarction. We also discuss the development and function of the cardiac lymphatic vasculature and how some immune cells interact with the lymphatic endothelium in the heart. Finally, we establish how promoting lymphangiogenesis is now a prime therapeutic target for reducing immune cell persistence, inflammation, and oedema to restore heart function in ischaemic heart disease.

## 1. Introduction

The lymphatic vasculature is a vital component of the cardiovascular system, consisting of a blind-ended, highly permeable vascular network, integral in maintaining tissue homeostasis, regulation of interstitial fluid, lipid absorption, fluid drainage, and immune cell trafficking [1,2]. Its role in immune cell transport is critical in the initiation of the immune response, especially following injury. This is of particular importance in the heart, where the lymphatic vasculature plays a vital role in myocardial healing following cardiac injury [3]. By promoting cell egress or exit from the heart, the lymphatic systems favour cell clearance by way of reduction of the immune cell load in damaged tissue. It is not yet fully understood how targeted this process is and whether it encompasses all cells in the interstitial space versus specific cell types. In this review, we will describe the role of immune cells in response to myocardial infarction (MI), lymphatic development, and its key role in modulating immune cell clearance post-MI, and finally, we will explore the therapeutic potential of targeting immunomodulation via the cardiac lymphatic system and future directions.

## 2. Immune Cells in the Heart

In this first part of the review, we introduce the major immune cell populations that infiltrate the heart following MI. We focus on their timelines and their functions during injury to better understand their roles, why their presence is important, and how they can also be deleterious for heart repair. This is summarised in Table 1.

### 2.1. Neutrophils: The First Cells to Arrive at the Site of Infarction

Within a few hours post-MI, the necrosis of cardiomyocytes and the consequent release of cell debris generates endogenous agents known as damage-associated molecular patterns (DAMPs) [4,35,36] which activate and recruit neutrophils to the site of infarction via their Toll-like receptors [7,37]. Neutrophils are defined as CD45^+^CD11b^+^Ly6C^+^ cells, but different subsets exist. As with other immune cells such as macrophages, neutrophils can be subcategorised into N1 and N2 subpopulations depending on their pro-inflammatory or anti-inflammatory profiles respectively [7]. At an early-stage post-MI (Day 1), N1 neutrophils (Ly6G^+^CD206^−^) exhibit high expression of pro-inflammatory markers, generate high levels of reactive oxygen species and initiate local inflammation as well as tissue destruction. By phagocytosis, they clear debris and dead cells in the infarcted myocardium [6]. From Day 5 post-MI, an N2 neutrophil population (Ly6G^+^CD206^+^), with an anti-inflammatory phenotype, begins to increase and plays an important role in the resolution of the infarct wound [7]. These complementary but opposing roles for neutrophils post-MI are necessary for heart repair and must be tightly regulated. Neutrophil depletion in a mouse model of MI worsens cardiac function and increases fibrosis. Interestingly, in the absence of neutrophils, the number of monocytes recruited to the infarct zone is reduced, whereas the number of macrophages is increased [8]. Moreover, the neutrophil gelatinase-associated protein lipocalin, secreted by neutrophils, polarises macrophages towards a reparative phenotype [8]. Paradoxically, the absence of neutrophils is deleterious; however, elevated neutrophil counts in ST-segment elevation myocardial infarction (STEMI) show a positive correlation with myocardial infarct size [38]. Thus, timely clearance of neutrophils from the infarct zone, may present a tractable therapeutic approach to improve heart recovery post-MI.

### 2.2. Monocyte/Macrophages: Cells Involved in Both Inflammation and Its Resolution

Monocytes and macrophages dominate the innate immune response post-MI in terms of sheer numbers and are the best studied leucocyte population in this context to date. Macrophages can be broadly categorised as either tissue-resident cells that emerge during embryonic development or monocyte-derived cells that derive from bone marrow and splenic reservoirs [39]. Tissue-resident macrophages originate from the yolk sac or foetal liver progenitors [40,41] and represent up to 8% of the total non-cardiomyocyte population in the healthy adult mouse heart [42]. In the embryonic mouse heart, they mediate remodelling of the coronary plexus and lymphatic endothelium, and thus appear as indispensable for development of the mammalian cardiac vasculature [43,44]. In adults, their primary role is to maintain homeostasis of the myocardium in the steady-state heart by removing senescent and dying cells and facilitating electrical conduction [12]. In addition, they are involved in the first steps of the inflammatory response following MI. Through a myeloid differentiation primary response-88 (MYD88)-dependent pathway, tissue-resident C-C motif chemokine receptor 2-positive (CCR2^+^) macrophages are responsible for the initial recruitment of monocytes to the infarct zone and the promotion of neutrophil extravasation [13,14]. Even if the resident macrophages are rapidly replaced by circulating monocytes, their genetic deletion decreases cardiac output and contributes to adverse remodelling, underlying their crucial cardioprotective role [15]. During the first phase of inflammation, high levels of monocyte chemoattractant protein-1(MCP-1, a CCR2 ligand) in the myocardium triggers the infiltration and accumulation of Ly-6C^high^ monocytes into the damaged tissue from the first day post-injury [9]. Ly-6C^high^ monocytes peak between Day 3 and Day 5 post-MI, and display high phagocytic and proteolytic activities, and express proinflammatory cytokines including tumour necrosis factor-α (TNFα) [10]. Genetic deletion of Ly-6C^high^ monocytes in *Ccr2^−/−^* mice impairs cardiac function and leads to higher elastin levels in the fibrotic scar tissue [11]. From Day 5 onwards, Ly-6C^low^ monocytes accumulate and trigger reparative processes including angiogenesis, by expressing high levels of vascular endothelial growth factor (VEGF), and inducing deposition of extracellular matrix (ECM) [10]. Up-regulation of macrophage-colony stimulating factor (MCSF) in the pro-inflammatory environment of the myocardium induces monocyte differentiation into macrophages [45].

In the mouse, macrophages differ from monocytes by virtue of increased F4/80 expression, while in the human they display increased expression of CD68 and the Major Histocompatibility Complex II (MHC II) and decreased expression of CD14 [46]. As for monocytes, two main subsets of macrophages occur in both species post-MI. Historically, macrophages have been divided into pro-inflammatory (M1) and anti-inflammatory or pro-resolving (M2); however, the reality is more complex as there is great heterogeneity in macrophage populations post-MI [47]. The early stage of MI resolution is characterised by the presence of pro-inflammatory M1 macrophages to promote clearance of matrix and debris through phagocytosis. Those pro-inflammatory macrophages produce various cytokines including interleukin (IL) -1β (IL-1β), IL6, and TNFα to sustain the inflammatory environment and to activate resident fibroblasts. As a result, several matrix metalloproteinases (MMPs) are released into the micro-environment and induce further ECM degradation [16]. This process is essential and must be tightly regulated to ensure an optimum environment before the resolution phase. The absence of this step or a prolonged inflammatory response leads to extensive damage and poor healing [17,48]. As M2 macrophages are reparative, they express anti-inflammatory cytokines (e.g., IL10). By secreting important growth factors, such as VEGF and transforming growth factor -β (TGF-β), they promote cell proliferation, angiogenesis, and ECM production respectively. TGF-β controls the expression of alpha smooth muscle actin (α-SMA) in the resident fibroblasts which drives their differentiation into myofibroblasts [49]. Myofibroblasts are the primary source of ECM proteins to replace myocyte loss and form a reparative scar [50]. However, it has been shown recently that macrophages themselves can also contribute to collagen deposition, and thus also fibrosis, during heart repair [18]. Deficiency of the pseudokinase Tribbles homolog 1 (Trib1) impairs the ability to form M2 macrophages without affecting the other immune cells [51]. *Trib1^−/−^* mice, which exhibit a selective depletion of M2-like macrophages, present a disastrous prognosis following MI. These mice display regular cardiac rupture due to reduced collagen fibril formation and by extension, poor infarct repair [19]. In contrast, depleting cardiac inflammatory monocytes and shifting macrophages towards the anti-inflammatory phenotype in the heart post-MI enhances cardiac recovery and plays a cardioprotective role [52]. Similarly, inducing an M2 phenotype in the infarcted heart through administration of IL-4 enhances cardiac function in conjunction with diminished infarct size and intensified tissue repair. This presented as reinforced connective tissue structure formation, enhanced microvascular growth, and impaired cardiomyocyte hypertrophy [53]. Many other approaches, including transplantation with human umbilical cord blood mesenchymal stem cells [54] or injection of exosomes from adipose-derived mesenchymal stem cells [55], have been used to promote M2 macrophage polarisation post-MI and resulted in improved cardiac tissue repair in MI models (see review [56]).

### 2.3. Dendritic Cells: Regulators of Immune Tolerance during MI

Dendritic cells (DC) play an important role at the intersection of the innate and adaptive immune systems in injured tissues including the heart [57]. They are the most efficient antigen-presenting cells, and their main function is to activate naïve lymphocytes. In common with monocytes and macrophages, they express high levels of MHC II and CD11c. In mice, DCs infiltrate the heart from day 1 post-MI, peak at day 5 [4], and subsequently migrate to the mediastinal lymph nodes (MLNs) where they can activate T-lymphocytes [21]. A recent study also shows that a cross-priming DC population is present and activated in the heart following MI. This population activates cytotoxic CD8^+^ T cells and their deletion reduces myocardial immunopathology and functional decline [58]. DCs can be classified into two major subpopulations: conventional DC (cDC) and plasmacytoid DC (pDC). Interestingly, selective deletion of cDC, using the zinc finger and BTB domain containing 46 (*Zbtb46)* promoter [59] in mice post-MI, improves cardiac function and prevents adverse cardiac remodelling. This is accompanied by a decrease in fibrosis in the infarcted zone and an attenuation of the inflammatory state due to the reduction of the number of immune cells including macrophages, neutrophils, and T cells [20]. In contrast, selective deletion of pDC, using the specific promoter *Bdca2* [60], does not affect heart function post-MI [20]. Total depletion of DCs promotes inflammation of the heart by increasing the infiltration of Ly-6C^high^ monocytes and M1 macrophages and impairing the recruitment of Ly-6C^low^ monocytes and M2 macrophages to the infarcted myocardium. As a result, deterioration of left ventricular function and remodelling was observed [22]. In line with these observations, the presence of small numbers of DCs in infarcted myocardial tissue in humans is associated with increased macrophage infiltration, impaired reparative fibrosis, and an increased risk of cardiac rupture post-MI [61]. By contrast, injections of tolerogenic DCs (toDC) in mice 24 h and 7 days post-MI induces infarct-specific regulatory T cells (T_reg_) in the mediastinal lymph node and promotes an inflammatory-to-reparative macrophage shift in the myocardium [23]. Moreover, generation of toDCs with IL-37 and troponin 1 increases the number of T_regs_ in vitro and in the spleen in vivo. Their injection attenuates the infiltration of inflammatory cells in the infarcted hearts [24], decreases myocardial fibrosis, and improves cardiac function and survival post-MI [23,24].

Taken together, these studies underline the protective role of DCs in post-MI inflammation and as a result, the healing process. It is important to highlight that the beneficial role of DCs occurs through the recruitment and activation of other cells outside the injury zone (e.g., within the MLNs and spleen) and consequently, the lymphatic network may play a crucial role in this process.

### 2.4. T-Lymphocytes: CD4^+^ Helper T Cells

T-lymphocytes are one of the main components of the adaptive immune response. They are divided into helper (CD4^+^) T-cells and cytotoxic (CD8^+^) T-cells. In the permanent coronary occlusion model of MI, CD4^+^ T-cells infiltrate the heart rapidly and peak at day 7 [4]. CD4^+^ knockout (KO) mice exhibit an increase in leucocytes and pro-inflammatory monocytes within the infarcted myocardium as well as impaired collagen matrix formation in the infarct zone, highlighting the significant role of CD4^+^ T-cells in myocardial wound healing [25]. In contrast, in the myocardial ischaemia/reperfusion (I/R) model, T-cells infiltrate the infarcted zone within minutes of injury and CD4^+^ T-cells contribute to the infarct size [26]. CD4^+^ T-cells can be further subdivided into different subsets, among them, Th1, Th2, and T_reg_ according to their phenotype, cytokine production, and function. The Th1 population is characterised by expression of the transcription factor T-bet and production of interferon-γ (INF-γ). The expression of dectin-2, a C-type lectin receptor, is highly upregulated in macrophages and neutrophils after one day post-MI and engagement with its ligand on T-cells polarizes them towards a Th1 phenotype. *Dectin-2* KO mice show an improvement in cardiac function post-MI, by promoting wound healing and scar formation, indicative of a detrimental role for Th1 cells in this process [62]. Moreover, a high Th1/Th2 ratio in patients with acute myocardial infarction correlates with an increase in adverse cardiac events [63].

The T_reg_ subset of CD4^+^ T-cells have immunosuppressive functions, and several studies have highlighted their beneficial impact on the heart post-MI. These cells express the lineage specification factor Foxp3 [64] and their numbers increase post-MI in mice to a peak at day 7. Genetic depletion of T_regs_ using *Foxp3DTR* mice leads to an increase in M1-like macrophages, resulting in aggravated cardiac inflammation and poor overall outcome. By contrast, T_reg_ activation induces M2-like macrophage differentiation, contributes to inflammation resolution, and improves wound healing and clinical outcome [27,28]. In another study, the adoptive transfer of T_reg_ had similar effects, reducing the infarct size and increasing the number of proliferating cardiomyocytes. Surprisingly, these beneficial effects are greater in the permanent ligation of left anterior descending artery (LAD) model than the I/R model [29]. The former model induces a large scar and significant apoptotic cell death and is appropriate to study wound healing whereas the latter, which creates a temporary occlusion leading to a smaller scar but causes a second wave of necrotic damage and is used for the study of reperfusion injury [65] which could explain the disparity in the results. Finally, in a rat model of MI, T_reg_ transfer decreased the recruitment of neutrophils, macrophages, and T-cells in the infarcted heart as well as the mRNA levels of pro-inflammatory cytokines [66]. Furthermore, the CD8^+^ cytotoxic T-cell response was impaired and the cardiac function improved [66]. Interestingly, the administration of a super-agonist anti-CD28 antibody to induce T_reg_ expansion led to similar results [66]. In mice, inhibition of the C-X-C motif chemokine receptor (CXCR) 4 (CXCR4) has been previously shown to improve cardiac function post MI [67] and pharmacological CXCR4 blockade promote myocardial repair by increasing T_regs_ function in vivo [68]. In addition, through the expression of cell surface lymphotoxin alpha beta (LTα1β2), T_regs_ activate the LT beta receptor (LTβR) on the lymphatic endothelial cells (LECs) to modulate their permissiveness and thus the transendothelial migration of leucocytes and by extend the resolution of inflammation [69].

In summary, by modulating inflammation, the T_reg_ population confers a protective role post-MI and presents a therapeutic opportunity for management of patients recovering from a heart attack. However, in a human phase 1 trial, the use of a super-agonist anti-CD28 antibody to induce T_reg_ expansion appeared to be highly toxic and caused a cytokine storm [70]. Hence, more physiological approaches for modulating cardiac inflammation such as clearing the immune cells via the lymphatic system, may offer an alternative and more successful approach to cardiac repair.

### 2.5. T-Lymphocytes: CD8^+^ Cytotoxic T Cells

The role of CD8^+^ T-cells in the context of acute heart ischaemia remains poorly understood. CD8^+^ T cells are recruited early to the ischaemic tissue, reaching a peak at 3 days post-MI [30]. *CD8^atm1mak^* mice, which are deficient in CD8^+^ T-cells, show an increase in immune cell numbers at day 3, a higher survival rate, and improved cardiac physiology as assessed by echocardiography on day 7 post-MI. However, due to poor scar formation, these mice undergo subsequent cardiac rupture, suggesting that CD8^+^ T-cells may have both beneficial and detrimental effects on heart recovery post-MI [31]. Moreover, specific immuno-depletion of CD8^+^ T-cells 1h after coronary ligation decreased infarct size and fibrosis and improved heart function, confirming the deleterious impact of such lymphocytes in the context of MI. Notably, these cells express the serine protease granzyme B, a component of cytotoxic granules, and studies targeting its disruption have observed the same beneficial effect on post-MI cardiac repair as total CD8^+^ T cell depletion. Interestingly, CD8^+^ T-cells and granzyme B^+^ cells were detected in human heart biopsies from acute MI patients and furthermore, high circulating levels of granzyme B have been shown to correlate with a higher risk of death [30].

### 2.6. B-Lymphocytes

Unlike T-cells, the roles of B-cells, the second major components of the adaptive immune system have been poorly characterised in MI. In mice, B-cells infiltrate the ischaemic cardiac tissue and peak at day 5 post-MI. These cells produce a diverse range of cytokines and chemokines including CCL7, a chemokine that contributes to myocardial inflammation by promoting the mobilisation and infiltration of circulating monocytes. Conversely, B-cell depletion reduces infarct size and improves cardiac function post-MI [32] and B-cells have been further implicated in collagen metabolism within the myocardium and impairing the left ventricular ejection function [33]. A recent study also revealed that pirfenidone, a drug bearing anti-inflammatory and anti-fibrotic properties, has a protective effect on myocardial infarction by modulating cardiac B-cell. Notably, pirfenidone was shown to block B-cell infiltration in the myocardium and prior depletion of B-cells abrogated the beneficial effects of the drug [34]. Hence, strategies aimed at accelerating the clearance of B-cells post-MI may be a potential way to improve heart remodelling and function.

In summary, immune cell infiltration and its functional consequences are the primary cause of inflammation of the myocardium during the acute stages of MI. Although such immune cells are clearly beneficial for tissue repair, clearance of necrotic cells, and tissue debris in the early stages of recovery, their prolonged presence impairs cardiac healing and has a deleterious effect on cardiac function through fibrotic scarring. Hence, promoting timely clearance of these cells from the site of injury has the potential to be an effective therapeutic approach for improving heart recovery post-MI. As will be discussed in the following section, the clearance of immune cells from the injured heart is heavily dependent on the cardiac lymphatic network.

## 3. Cardiac Lymphatic Network

### 3.1. Lymphatic Development in the Embryo

Under steady-state conditions the lymphatic system functions as normal. However, under pathological conditions, the lymphatic vasculature undergoes remodelling akin to what is seen during development. Therefore, understanding the development of the cardiac lymphatic system is essential in understanding its role in health and disease, and has previously been reviewed [71]. The lymphatic network arises both by budding from the blood vasculature and independently from lymphangioblasts during embryogenesis, in a process that is highly conserved in vertebrates. In the first instance, blood vessels are formed *de novo* through vasculogenesis from mesoderm- and somite-derived progenitors to form a primitive vascular network, which then matures through angiogenesis [72]. Subsequent to the formation of this vascular network, between embryonic days 9.5–10.5 (E9.5–E10.5) in mice, endothelial cells (ECs) in the cardinal vein (CV) then begin to express the lymphatic marker and master regulator of lymphatic endothelial cell specification Prospero-related homeobox domain 1 (PROX1) [73,74]. It is now clear that PROX1 is essential for the specification and ongoing development of the lymphatics, as *Prox1* KO mice lack lymph sacs and lymphatic vessels [73] and show embryonic lethality. Furthermore, the endothelial-specific knockout of *Prox1* results in lymphatic defects and postnatal lethality [75] and *Prox1* overexpression is sufficient to direct ECs towards a lymphatic fate both in vitro [76] and in vivo [77], further emphasising its importance in lymphatic development. The PROX1^+ve^ lymphatic endothelial cells (LECs) then bud from the cardinal vein and form primitive lymph sacs in response to the binding of VEGF-C [78] to its cognate, lymphatic endothelium-specific receptor, VEGF receptor 3 (VEGFR3). *Vegf-c* KO mice lack lymphatic vessels [78] and show embryonic lethality. Furthermore, *Vegf-c^+/−^* mice develop cutaneous lymphatic hypoplasia and lymphoedema, which can be rescued by VEGF-C treatment [78]. The nascent lymphatic network undergoes branching and network formation throughout later stages of development and postnatally, regulated by angiopoietin 2 (Ang2) and its receptor, TIE2. Ang2 KO mice show defects in lymphatic vascular remodelling [74], indicating that Ang2 may not be necessary for lymphangiogenesis de novo, but rather is critical for subsequent branching and pruning of the lymphatic vascular network.

In mice, a population of cardiac PROX1 and VEGFR3 expressing LECs originate from extra-cardiac tissue and the CV at E10.5 and migrate to the outflow tract and sinus venosus by E12.5, where they expand to form the cardiac lymphatics [79]. Much like the systemic lymphatics, the cardiac lymphatics follow the lead of the blood vascular system, and only appear following the formation of the sinus venosus, but before the onset of the coronary circulation [79]. Interestingly, the cardiac lymphatic vessels display significant heterogeneity, as lineage tracing identified that only 80% of cardiac LECs are venous-derived [79] with the remaining non-venous derived LECs, known as lymphangioblasts, originating from other sources including the haemogenic endothelium and are initially PROX1 deficient [80]. By E14.5, LECs mature and express lymphatic lineage markers including the LYmphatic Vessel Endothelial hyaluronan receptor 1 (LYVE-1) and the sialoglycoprotein podoplanin, by which point the cardiac lymphatics progressively extend from the base towards the apex of the heart, covering most of the developing organ [79,80]. Cardiac lymphangiogenesis is also dependent on VEGF-C and VEGF-D [78]. Transgenic mice expressing soluble VEGFR3 showed perturbed lymphatic development in the heart and other organs and also developed severe oedema and pericardial fluid accumulation [81]. Given that the epicardium and outflow tract is also a source of VEGF-C, they may be orchestrators of VEGF-C signalling and lymphatic sprouting [82]. Cardiac lymphatic vessel maturation and remodelling in mice continues postnatally for the following 2–3 weeks after birth [79,83]. Once fully matured, in humans, the lymphatic vasculature reaches all layers of the heart, including the atria, ventricles, and mitral valves [84]. In mice, the cardiac lymphatics extend predominantly around the branches of the coronary arteries and veins [85,86] and reside predominantly within the outer myocardium and compact wall of the chambers. Histological analysis at multiple developmental time points revealed that there are two pre-collector vessels. The left major pre-collector vessel runs along the left conal vein and under the left auricle towards the nearest lymph nodes. The second pre-collector runs parallel to the left cardiac vein toward the coronary sinus where extracardiac larger collector vessels empty into draining mediastinal lymph nodes which are found beneath the aortic arch and around the trachea [84,87]. Interestingly, mouse and human cardiac pre-collectors, unlike those of most other tissues contain very few smooth muscle cells [88,89]; thus, lymph propulsion from the heart appears to be dependent on extrinsic factors such as cardiac muscle contraction. Therefore, instances where cardiac contractility or heart rate are compromised, affect cardiac lymph flow accordingly [90,91].

### 3.2. Cardiac Lymphatic Remodelling Following Myocardial Infarction

**Figure 1:**
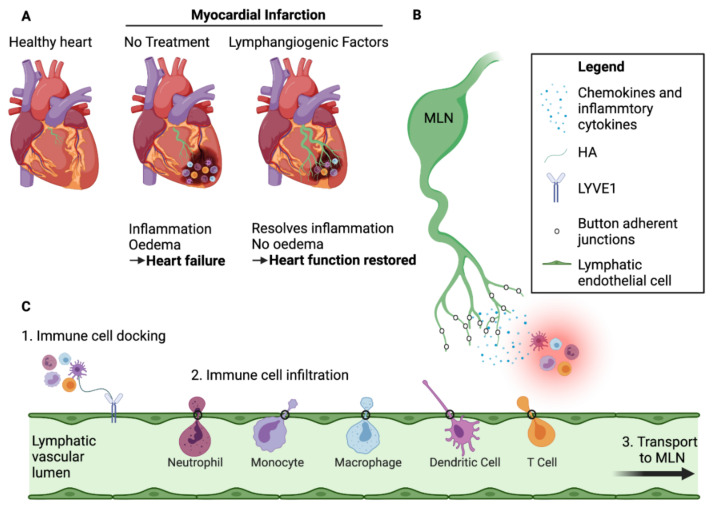
Lymphangiogenesis improves cardiac function post-MI. (**A**) Schematic comparison between a healthy heart and a heart subject to myocardial infarction (MI) with or without lymphangiogenic growth factor treatment. Following MI, immune cells infiltrate the infarct zone and create an inflammatory environment that impedes healing of the injured heart. Lymphangiogenesis occurs in non-treated hearts due to endogenous VEGF-C signaling, but is not sufficient to clear the immune cells and avoid myocardial oedema, resulting in scar formation and heart remodelling. Treatment with lymphangiogenic factors, such as VEGF-C(C156S) which binds exclusively to VEGFR3, greatly augments the lymphangiogenic response leading to efficient clearing of excess tissue fluid and pro-inflammatory cells, resulting in less severe heart remodelling and improved cardiac healing and function. (**B**) Cross-talk between lymphatic capillaries and immune cells in the infarcted area. Lymphatic endothelial cells secrete chemokines including CCL21, C-X3-C motif chemokine ligand 1(CX3CL1), and CXCL12, which attract specific populations of immune cells according to their receptor expression profile. These endothelial cells also express adhesion molecules, such as ICAM-1 and VCAM-1, which facilitate immune cell crawling. (**C**) Steps involved in immune cell clearance by the lymphatic vessels. Through their assembly of an endogenous HA surface glycocalyx, immune cells dock with LYVE-1 homodimers in the button-like endothelial junctions of initial afferent capillaries and enter the vessel lumen to migrate towards downstream MLNs. Created with BioRender.com.

Lymphatic remodelling is a process typically seen only during embryonic development or following injury or disease. In the heart, lymphatic remodelling can occur in both acute and chronic heart failure [81,92,93], following cardiac transplantation [94], and in atherosclerosis [95]. In recent years, research into enhancing lymphangiogenesis in the context of heart disease has become a key area of clinical interest. Cardiac lymphatic remodelling is often investigated in the context of MI in mice. One of the myriad consequences of MI and prolonged ischaemia is the death of endothelia, owing to increased vascular permeability and loss of lymphatic vessels, both of which result in poor fluid drainage and prolonged oedema [89,96]. In mammals, this loss of cardiovascular tissue results in heart remodelling, such that the dead myocardium is replaced with scar tissue. In an attempt to resolve the oedema, the cardiac lymphatics also undergo remodelling in the form of lymphangiogenesis in the infarct zone [10,89,97,98] (Figure 1). In parallel, following MI, there is an increase in local inflammatory cytokine and chemokine production, resulting in increased activation and recruitment of innate immune cells to the infarct area as discussed above [99]. Inadequate lymphangiogenesis results in increased interstitial fluid and osmotic pressures, which further amplify local immune responses through activation of osmoregulatory mechanisms in resident immune cells [100]. Moreover, inadequate lymphangiogenesis also results in the persistence of immune cells in the infarct region, as a result of their reduced clearance to draining cardiac lymph nodes. Ultimately, the events described result in fibrosis, impaired heart function, and eventually heart failure [90,96]. Furthermore, during the chronic phase following MI, the lymphatic remodelling of pre-collectors results in poor cardiac lymph transport leading to chronic myocardial oedema [17,79,89]. More details on the role of lymphangiogenesis post-MI will be discussed in the final section of this review.

### 3.3. Exit of Immune Cells from the Infarcted Heart—The Role of Cardiac Lymphatics

In addition to regulating interstitial fluid homeostasis, the lymphatics are an integral component of the immune system, facilitating the transport of immune cells, pathogens, and antigens [101] from the sites of injured and infected tissues to draining lymph nodes (dLNs) for generation of protective T and B cell responses [101,102]. Allied to this, they also provide a key route for exit of immune cells during the resolution of tissue inflammation, not only following myocardial infarction, but also lung injury and allograft rejection [97,103,104,105]. In normal resting tissue the numbers of immune cells migrating in afferent lymphatics are small, comprising mainly T-cells (approximately 90%) and immature antigen presenting dendritic cells (DCs) engaged in background immune surveillance. However, in injury and inflammation the numbers of immune cells in afferent lymph rise several-fold, due to an upsurge in lymph flow, an increase in lymph vessel permeability and the local release of pro-inflammatory cytokines [106]. The migrating populations include recirculating antigen-experienced memory T-cells (T_RCM_), immunoregulatory T-cells (T_reg_) and small numbers of B-cells that patrol the tissues for cognate antigen, mature DCs ferrying internalised antigens for immune priming, and macrophages and neutrophils involved in pathogen killing, clearance of tissue debris and tissue repair/remodelling [97,107,108,109,110]. The trafficking of each cell population is carefully choreographed, with antigen-charged DCs normally being the first to enter the lymphatics from the tissues. However, neutrophils are the most rapidly mobilised immune cells, and in some contexts (e.g., post-vaccination) the first to migrate to dLNs in inflammation, arriving some 72 h before either DCs or macrophages [111,112] (see Table 1). Of note, afferent lymph contains few if any naïve T or B cells, as these are absent from resting tissues. Instead, they enter LNs directly from the blood via high endothelial venules (HEVs) and recirculate via efferent lymph through the thoracic duct and subclavian veins [113].

The initial afferent vessels through which the above-mentioned immune cells enter the tissue lymphatics begin as blind-ended capillaries with a distinctive architecture composed of oakleaf-shaped LECs joined together by loose, discontinuous junctions that lack a substantial basement membrane [114]—characteristics well suited to a role in fluid drainage. Such junctions operate as primary valves that allow the one-way entry of fluid to the vessels while preventing its backflow to the interstitium [115,116]. Importantly, the interdigitating arrangement of oakleaf shaped endothelial cells creates a succession of overlapping flaps, and these are buttoned at their sides by adherens junction and tight junction proteins including VE-cadherin, JAMs, claudins and ESAM, while more loosely attached at their tips by the homotypic Platelet Endothelial Cell Adhesion Molecule, PECAM-1 (a.k.a. CD31), and the HA receptor LYVE-1 [117]. Notably, as revealed by electron microscopy and high-resolution confocal imaging, the alternating flaps guard openings of ~0.5–1µm in size [118] that act as portals for migrating DCs and macrophages which enter the afferent lymphatics by a process of pushing and squeezing [118,119,120]. Moreover, as discussed below, the discrete location of LYVE-1 at these portals is fully consistent with the key function of the receptor in mediating such immune cell entry.

Downstream of initial lymphatics, the larger pre-collector and collector vessels are more tightly sealed by conventional tight “zipper”-like junctions, akin to those of blood vessels. In keeping, their constituent LECs have a more regular rather than oakleaf shape and express far lower levels of LYVE-1 [117,118]. In addition, the pre-collector/collectors are invested by smooth muscle cells, whose contraction enables the conveyance of leukocytes to dLNs via lymph flow [121]. Notably, lymphatic vessels present in embryonic tissue have exclusively zippered junctions, and only transition to the button-like junctions of initial capillaries late in development and during the early neonatal period. Moreover, in chronic inflammation and tissue injury, the lymphatics also display significant junctional plasticity, such that new vessels generated during lymphangiogenesis as well as surrounding pre-existing vessels have zipper-like junctions similar to those of early embryos [117].

### 3.4. Mechanisms of Immune Cell Exit via the Cardiac Lymphatics

The exit of immune cells through cardiac lymphatics involves a series of consecutive steps that have been defined primarily from studies of DC and T-cell trafficking in inflamed mouse skin [106,122]. The first of these is the passage through the interstitium to reach the initial afferent lymphatic capillaries, a process that relies on chemotaxis and amoeboid migration and is largely independent of integrin-based adhesion [106]. Proceeding at background levels in normal tissues as already described, such interstitial migration is upregulated in inflammation by local release of cytokines including IL-1, IL-18, TNFα, and IFNγ that promote immune cell migration to dLNs [123,124] and in the case of dermal DCs, their mobilisation and differentiation to a more motile state, pre-adapted for antigen uptake and presentation by class II MHC molecules. Importantly, these same cytokines induce local secretion of CCL21 from the lymphatic endothelium, the single most important chemokine in control of DC, macrophage, and T cell trafficking via lymphatics [109,125,126], a process further facilitated by the increase in interstitial fluid flow that accompanies tissue injury [125,126,127]. CCR7, the signal transducing receptor for CCL21 is critical for such chemotaxis, as evidenced by the finding that DCs deficient in CCR7 show a 90% reduction in migration from the periphery to dLNs in response to injury [128]. Other chemokines and chemokine receptors involved with immune cell trafficking include CCL2 [126,129], C-X-C motif chemokine receptor (CXCR) 3 (CXCR3) [130], and CXCR4 [129]. In addition, inflamed lymphatic endothelium also secretes a variety of other chemokines, including CCL2, CCL5, CCL20, CXCL2, IL-8 and CX3CL1 (fractalkine) that direct selective exit from inflamed tissues of T- cells, monocytes, macrophages and neutrophils bearing the appropriate receptors [126,127,129,130,131].

The second step, which is rate-limiting to immune cell exit, is adhesion to the basolateral surface of lymphatic vessel endothelium and transmigration to the vessel lumen. As established by recent and ongoing research based on DCs, this involves early interaction between the large glycosaminoglycan hyaluronan (HA), anchored within the dense 500 nm glycocalyx of migrating immune cells by the leucocyte receptor CD44, and its cognate receptor LYVE-1 in the button-like junctions of capillary endothelium (see Figure 1) [118,132,133]. These initial contacts, aided by the extended dimensions of HA chains, trigger the formation of LYVE-1 dense, cup-like membrane protrusions in the underlying LECs that extend around the adherent leucocytes, shepherding their transit across the endothelium [133]. Moreover, LYVE-1, CD44 and HA are each critical both for assembly of transmigratory cups and for subsequent transendothelial migration, as either genetic deletion of the receptors or enzymatic digestion of the bound glycocalyx blocks trafficking of DCs to dLNs in mice [132,133]. As deduced from confocal and video-microscopy, CD44 not only tethers the HA glycocalyx to the immune cell surface but is also responsible for its actin-mediated re-distribution to the uropod, the membrane protrusion at the posterior pole of the cell that co-ordinates LYVE-1 binding and endothelial adhesion [132,134].

Following transmigratory cup formation, the transit of immune cells across lymphatic endothelium involves the participation of numerous additional adhesion receptors, in particular β1 and β2 integrins and their respective counter-receptors VCAM-1 and ICAM-1. Notably, both are highly upregulated in dermal LECs in response to contact hypersensitising agents and inflammatory cytokines including IL-1 and TNFα both in vitro and in vivo, and ICAM-1 co-localises with LYVE-1 in transmigratory cups [131,133,135,136]. Furthermore, VCAM-1 is also expressed in downstream lymphatic collectors under such conditions, where it can mediate immune cell entry through conventional zippered junctions, likely supporting a second route for leucocyte exit, independent of LYVE-1, in chronically inflamed tissues [137].

Curiously, however, the mechanism by which neutrophils transit the lymphatic endothelium is quite distinct from that of other immune cells, as evidenced by studies both in vitro and in vivo using inflamed LEC monolayers and mouse models of trafficking in bacterial infection [138,139]. This process involves neither the assembly of a HA glycocalyx nor adherence to lymphatic endothelium via LYVE-1. Although initial attachment is mediated by neutrophil β2 integrins, the interaction induces co-ordinate release of neutrophil elastase, matrix metalloproteinases MMP8 and MMP9, and the arachidonate-derived chemorepellent lipid 12-hydroxyeicosatetraenoate (12(S)HETE) which together trigger endothelial junctional retraction and enable transit of the cells at a rate nearly 10-fold higher than that of DCs [138].

Just as in interstitial migration, the subsequent steps of adhesion and transendothelial migration that enable immune cell exit through afferent lymphatics are also guided and directed by chemokines. Again, this is mediated mainly by CCL21 which is secreted by LECs in the form of haptotactic gradients immobilised on peri-lymphatic heparan sulphate proteoglycans such as perlecan and on subendothelial collagen IV [140,141]. Upregulated in inflammation, CCL21, through its interaction with CCR7, triggers the conformational activation of immune cell β integrins, increasing their binding affinity for ICAM-1 and enabling diapedesis. Interestingly, the on-demand release of CCL21 for transmigration is elicited by physical contact between DCs and the underlying vessel endothelium. This interaction triggers the secretion of chemokine from pre-stored depots in *trans* Golgi vesicles by a Ca^2+^ triggered exocytic mechanism that involves transport by microtubules and fibrillar actin and that results in its deposition immediately adjacent to transmigrating DCs [142].The identities of the receptor(s) on immune cell and the corresponding ligand(s) on lymphatic endothelium that mediate CCL21 exocytosis, however, are currently unknown.

It will be clear from the foregoing discussion that afferent lymphatic capillaries and downstream collector vessels are critical routes for the exit of immune cells from injured and inflamed tissue to dLNs, and that transit from the surrounding interstitium to the vessel lumen is a tightly regulated and rate-limiting step in the process. Moreover, as is the case for strategies aimed at boosting lymphangiogenesis, augmenting the process of immune cell exit may yet prove to have potential as a target for therapy in MI.

## 4. Lymphangiogenic Therapy Post-MI

It is well known from studies in both humans and rodents that MI induces pathological remodelling of the cardiac lymphatics [79,89,92,143,144]. Indeed, the characteristic oedema in the myocardial interstitium that results from ischaemia is clearly indicative that lymphatic drainage is insufficient for fluid drainage. At early stages post-MI, histopathologic analysis of patients with acute MI reveals the progressive loss of lymphatic vessels from the interstitium, as compared with the normal myocardium. Nevertheless, studies using mouse models of myocardial I/R and MI have shown that normal lymphatic density is subsequently restored at later stages post-MI; in the subendocardial compartment, lymphatic density was significantly augmented 3 days post-MI and gradually increased until day 7 [92]. This augmentation is likely to be driven by VEGF-C, the main lymphangiogenic growth factor acting through VEGFR3 during embryonic development. VEGF-C is expressed in the cardiomyocytes around the cardiac lesion and as such may act as a source for the restoration of cardiac lymphatic vessels [144]. Additionally, macrophages and the epicardium represent another source of VEGF-C post injury [97,145] and neutrophils have been described as organisers of lymphangiogenesis during inflammation by increasing VEGF-A bioavailability and secreting VEGF-D [146]. However, in rats, despite the significant increase in the density of lymphatic capillaries at 4 weeks post-MI, the percentages of pre-collector vessels, as well as open lymphatics and their diameters were decreased, likely explaining the poor lymphatic draining capacity and the subsequent persistence of myocardial oedema [89]. The death of cardiomyocytes during MI reduces cardiac contractility, which in turn impedes lymph propulsion from the heart to MLNs [147], and may also be responsible for inefficient fluid drainage.

As discussed above, sub-optimal heart recovery following MI is mainly due to the persistence of immune cells in the infarcted zone that delay or prevent the resolution of inflammation and the timely repair of cardiac injury. Given that one of the primary functions of lymphatic vessels is immune cell clearance from injured tissue, several groups have investigated the importance of lymphangiogenesis post-MI. Post-MI treatment with VEGF-C(C156S), an artificially mutated form of VEGF-C that binds exclusively to VEGFR3, induces a lymphangiogenic response in the rodent heart that results in an improvement of cardiac function [79,89,92,97,148]. In a mouse model of MI, intraperitoneal (i.p.) injection of VEGF-C(C156S) augmented cardiac lymphangiogenesis after injury. Interestingly, in the treated group the numbers of infiltrating leukocytes, including macrophages and DCs were shown to be significantly reduced 7 days post-MI, indicating enhanced clearance of immune cells to the MLNs. On the contrary, LYVE-1 gene deletion was shown to worsen cardiac outcomes and to promote chronic inflammation, due to the reduced ability of Lyve1^−/−^ lymphatics to clear the immune cells [97]. Curiously, Houssari et al., using the same approach, failed to observe a significant increase of lymphangiogenesis after i.p injection of VEGF-C(C156S) [148]. However, it is important to note that these workers quantified cardiac lymphatic vessel density by conventional histology, whereas the former study used whole-mount LYVE1 immunostaining ([148] and [97] respectively) which could explain the discrepant findings. Moreover, cardiac function and heart remodelling post-MI after VEGF-C(C156S) treatment were not evaluated in the Houssari study, making a conclusion based exclusively on standard histology difficult to reconcile. Nevertheless, they also employed a different approach with an i.p injection of an adeno-associated viral vector encoding VEGF-C(C156S) (AAV-VEGF-C(C156S)) 7 days before MI and observed an increase in lymphangiogenesis 7 days post-MI as well as a decrease in both T-cells and pro-inflammatory macrophages in the viable left ventricle but not in the infarcted area 21 days post-MI. Fractional shortening was increased in mice treated with AAV-VEGF-C(C156S) therapy, indicating an improvement of cardiac function [148]. Another group adopted an intramyocardial, targeted delivery of VEGF-C(C156S) using albumin-alginate microparticles in a rat model. Here, high doses of the growth factor significantly increased the lymphatic density in the subepicardium by 3 weeks post-MI. 8 weeks post-MI, the frequency of larger epicardial pre-collectors was increased in treated rats and contributed to improved cardiac lymphatic drainage. 3 weeks post-MI, myocardial water balance was also improved, and the numbers of macrophages in the infarcted left ventricle were reduced significantly. MRI and echocardiography analysis confirmed the therapeutic lymphangiogenic effect on cardiac perfusion and function [89]. To further investigate this, another team used hydrogel as a strategy for VEGF-C(C156S) delivery. In a mouse myocardial I/R model, the gel was placed on the surface of the myocardium at the time of re-perfusion. Seven days later, the lymphatic density increased, and the number of B-cells decreased, as did myocardial oedema and the levels of various pro-inflammatory cytokines such as TNF-α, IL1β, and IL-6. 28 days post-reperfusion, the infarct scar, the LV end-diastolic diameter, and LV end-systolic diameter were all reduced, while the ejection fraction was improved in comparison with control mice, showing that hydrogel containing VEGF-C(C156S) can indeed limit heart failure. Conversely, inhibiting VEGFR3 or VEGF-C with a neutralising antibody exerted the opposite effect and aggravated cardiac dysfunction [92]. Thus, collectively there is now substantial evidence that delivery of VEGF-C(C156S) by a variety of routes in experimental animal models can significantly improve the outcome post-MI; through targeting increased lymphangiogenesis to reduce oedema and enhance the clearance of immune cells.

The half-life of VEGF-C is extremely short [149] which suggests it is not an ideal target for therapeutic use. Clinical trials using intramyocardial adenovirus vector-mediated VEGFD-ΔNΔC gene therapy in patients with refractory angina have established the safety and feasibility of this therapy, accompanied by a positive outcome in treated patients with an increase of the myocardial perfusion [150]. Although quite promising, the approach nevertheless remains invasive, with the necessity for repeated injections in the myocardium that have a high cost per patient. Hence, it will be necessary to find other means of manipulating lymphangiogenesis (growth factors, compounds, existing drugs) to gain a better understanding of the mechanisms involved, and to discover potential new treatments for heart repair.

One alternative strategy, overexpression of the epicardium-specific peptide, adrenomedullin, has recently been shown to trigger lymphangiogenesis post-MI and to improve cardiac function in mice. Interestingly, sex-dependent differences were noted, with a decrease in myocardial oedema that was found exclusively in males. Furthermore, ejection fraction and fractional shortening were improved after only 10 days in females versus 15 days in males. This indicates an important limiting factor in the discovery of novel lymphangiogenic compounds, as in the steady-state heart, cardiac lymphatic density also differs between males and females [151]. The chemoattractant Shingosine-1-phosphate (S1P) has been described as another lymphangiogenic mediator both in vitro and in vivo [152] and may have a role in lymphatic vessel maturation [153]. In addition, S1P has been well described as a lipid mediator of leucocyte egress from lymphoid organs [154]. Though it has also been implicated in DC trafficking [155], its role in the trafficking of immune cells from inflamed tissues to dLNs is poorly understood and warrants further investigations especially in the context of MI.

Finally, studies have also focused on cell-based therapies to increase lymphangiogenesis and restore heart function post-MI. Using a rat model of MI, Zhang et al. investigated the potential effects of transplanting lymphatic endothelial cell progenitors (LECP) either alone or in combination with VEGF-C, using a self-assembling peptide (SAP) hydrogel that facilitated a sustained release of the growth factor [156,157]. Individually, both treatment strategies led to an improvement in cardiac function and their combination yielded an additive effect by significantly reducing myocardial oedema and fibrotic scar size. Moreover, the numbers of infiltrating immune cells correlated inversely with the number of lymphatic vessels and both the ejection fraction, and the fractional shortening were restored in the treated rats [156]. Hence, this represents a feasible strategy for therapeutic use in the future. Cardiac fibroblasts are another cell type with potential beneficial properties for heart repair. In particular, a specific subpopulation expressing VCAM-1 (CFV) has been identified as a potential inducer of lymphangiogenesis. This population expresses several pro-lymphangiogenic factors, including VEGF-C, and was shown to promote lymphangiogenesis as assessed by assays for in vitro tube formation. Furthermore, injection of human foetal CFV in post-infarct heart failure rat models mobilised LECs into the infarcted area and restored cardiac contractility [158].

In addition to clearing immune cells and resolving myocardial oedema, it has been recently shown that cardiac lymphatics may confer other beneficial effects on heart recovery post-MI. LECs secrete a variety of growth factors, cytokines, and chemokines known as “lymphangiocrine” factors, that are active during the initiation of immune responses [159]. The LEC secretome contains the extracellular protein reelin which promotes cardiomyocyte proliferation and survival. Cardiac delivery of reelin post-MI, in mice, improves heart function by exerting a cardioprotective effect [160].

Additionally, lymphangiogenic therapy also has a positive outcome in other cardiac diseases. In human chronic heart failure, the levels of lymphatic endothelial markers are decreased in comparison with healthy donors [161]. Using the Ang2 infusion-induced mouse model, Song et al. demonstrated that co-administration of VEGF-C(C156S) prevented cardiac dysfunction due to an improvement of the cardiac lymphatic vascular function and a decrease of the inflammatory response. After one week, inflammatory and fibrosis markers were decreased, and after five weeks, hypertension in the VEGF-C(C156S) treated group was almost abolished [161]. This is important, since survival from acute injury event in MI patients is significant, however, subsequent progression to heart failure arising from pathological remodelling remains a major cause of morbidity and mortality for which the only cure is heart transplantation. Furthermore, advances in 3D imaging of the cardiac lymphatic vasculature in vivo in mice has identified that increasing lymphatic vascular density may not be enough to resolve inflammation and cardiac oedema [162]. Furthermore, advances in imaging in humans have also highlighted the importance of addressing lymph flow when addressing heart disease [163,164]. Therefore, ensuring there is increased lymph flow, in addition to enhanced lymphangiogenesis, is also critical in the trafficking of immune cells from the site of injury and may also be a potential therapeutic approach [162,163]. Targeting the lymphatics during the chronic phase post-MI, therefore, to potentially alleviate the major drivers of heart failure presents an attractive target for therapeutic lymphangiogenesis.

## 5. Conclusions

To date, significant effort has been directed towards discovering new anti-lymphangiogenic drugs to tackle diseases ranging from metastatic cancer, organ graft rejection, and lymphoedema [165]. In contrast, there is a distinct lack of novel therapeutic strategies aimed towards the promotion of lymphangiogenesis. In the context of MI or myocardial oedema, where the process is pivotal to inflammatory cell clearance, such strategies offer a promising interventional approach to optimise heart repair, reduce the incidence of heart failure and thus improve patient recovery and long-term prognosis. Currently, heart transplantation is the only long-term solution for patients developing heart failure post-MI, but one that is still confounded by host immune rejection, excessive cost and limited donor heart availability. Clinical trials using cell transplantation to potentially aid heart repair and restore lost cardiovascular tissue post-MI have had only modest and transient patient benefit with low positive outcomes [166,167]. This is almost certainly due to very poor cell engraftment and survival during the pro-inflammatory phase post-MI. Thus, modulating inflammation by triggering lymphangiogenesis to reduce the inflammatory and pro-fibrotic milieu might facilitate improved cell-based therapy in the future. Additionally, modulating the immune system post-MI by increasing M2-like macrophages and T_regs_ also represent another promising therapeutic strategy to promote cardiac repair [168].

Given that cardiac lymphatics are involved in many aspects of cardiac disease, fibrosis, and inflammation [88,92,93] their clinical significance warrants further study. It is now clear that lymphangiogenesis is a vital process for successful heart repair and represents a highly promising therapeutic target for reducing cardiac damage and improving heart function in patients post-MI. However, the complexity and diversity of immune cells mean that deciphering which cells are drained, when, and how, is still poorly understood. Whilst much is known about how DCs enter and migrate within the cardiac lymphatics, a similar understanding of such interactions among other key immune cell populations will be essential to furthering our knowledge of how lymphangiogenesis can be applied therapeutically.

## Figures and Tables

**Table 1:** Role of immune cells post-MI.

**Cell Type**	Subtypes	Markers	Timeline [4,5]	Function	KO Effect	References
Neutrophils	N1	CD45^+^CD11b^+^Ly6C^+^ Ly6G^+^CD206^−^	From Day 1 to day 5	Clear debris and dead cells. Pro-inflammatory	Neutrophil deletion worsens cardiac function and increases fibrosis *	[6]
	N2	CD45^+^CD11b^+^Ly6C^+^ Ly6G^+^CD206^+^	From Day 5	Anti-inflammatory		[7,8]
Monocytes	Inflammatory	CD14^+^Ly-6C^high^ CCR2^high^CX3CR1^low^	From the 1^st^ day and peak at day 3–day 5	Phagocytose and proteolytic activities. Inflammatory	Cardiac Fibrosis Heart Failure	[9,10,11]
	Non-Classical	CD14^+^Ly-6C^low^ CCR2^low^CX3CR1^high^	From day 5 onwards	Reparative process: Angiogenesis & ECM deposition	Acute inflammatory reaction 7 days post MI. No long-term differences in scar formation	[10]
Macrophages	Residents	CD45^+^CD11b^+^ Ly6G^–^F4/80^+^	Present before MI. Quickly replaced by other immune cells	Maintain homeostasis. Recruit monocytes and promote neutrophil extravasation post-MI	Adverse remodelling	[12,13,14,15]
	M1-Like	CD45^+^ Ly6G^–^CD11b^high^ F4/80^+^CD206^−^ Ly-6C^high^	Peaks at day 3	Phagocytose and proinflammatory	High mortality rate. Increase remodelling	[16,17]
	M2-Like	CD45^+^ Ly6G^–^CD11b^low^ F4/80^+^CD206^+^ Ly-6C^low^	Appear 3 to 5 days post-MI and onwards	Anti-inflammatory. Promote cell proliferation, angiogenesis, and ECM production	Cardiac rupture	[18,19]
Dendritic Cells	cDC	CD45^+^MHCII^+^ CD11c^+^Zbtb46^+^	Infiltrate at day 1 and peak at day 5 *			[20]
	pDC	CD45^+^MHCII^+^ CD11c^+^BDCA2^+^		Antigen Presentation to T-Lymphocytes *	Improves cardiac function & attenuation of inflammation	[21,22] *
	toDC	CD45^+^MHCII^low^ CD11c^+^		Activate Tregs	Total DC depletion promotes inflammation and increases cardiac rupture *	[23,24]
T- Lymphocytes	CD4+	CD45^+^CD11b^−^ CD3^+^CD4^+^	Infiltrate at day 1 and peak at day 7	Produce pro-inflammatory cytokines	Increase pro-inflammatory monocytes. Impair collagen matrix formation	[25,26]
	Tregs	CD45^+^CD11b^−^ CD3^+^ CD4^+^CD25^+^FoxP3^+^	Infiltrate at day 1 and peak at day 7	Promote inflammatory-to-reparative macrophage	Cardiac inflammation	[27,28,29]
	CD8+	CD45^+^CD11b^−^ CD3^+^CD8^+^	Gradually increase post-MI	Removal of necrotic tissue. Cytotoxic effect	Infarct size and fibrosis decreased. Heart function improved but cardiac rupture	[30,31]
B- Lymphocytes	N/A	CD45^+^CD11b^−^ CD3^−^CD19^+^	Peaks at Day 5–7	Monocyte mobilization Sustain myocardial inflammation	Reduce infarct size and improve cardiac function	[32,33,34]

* not specific to a subset but apply to the whole population.
